# Microwave Heating-Assisted Catalytic Dry Reforming of Methane to Syngas

**DOI:** 10.1038/s41598-018-27381-6

**Published:** 2018-06-12

**Authors:** Sepehr Hamzehlouia, Shaffiq A. Jaffer, Jamal Chaouki

**Affiliations:** 1 Department of Chemical Engineering, Polytechnique Montreal, c.p. 6079, Succ. Centre-ville, Montreal, Quebec, H3C 3A7 Canada; 2 Total American Services, Inc., 82 South St., Hopkinton, MA 01748 USA

## Abstract

Natural gas is a robust and environmentally friendlier alternative to oil resources for energy and chemicals production. However, gas is distributed globally within shales and hydrates, which are generally remote and difficult reserves to produce. The accessibility, transportation, and distribution, therefore, bring major capital costs. With today’s low and foreseen low price of natural gas, conversion of natural gas to higher value-added chemicals is highly sought by industry. Dry reforming of methane (DRM) is a technology pathway to convert two critical greenhouse gas components, CH_4_ and CO_2_, to syngas, a commodity chemical feedstock. To date, the challenges of carbon deposition on the catalyst and evolution of secondary gas-phase products have prevented the commercial application of the DRM process. The recent exponential growth of renewable electricity resources, wind and solar power, provides a major opportunity to activate reactions by harnessing low-cost carbon-free energy via microwave-heating. This study takes advantage of differences in dielectric properties of materials to enable selective heating by microwave to create a large thermal gradient between a catalyst surface and the gas phase. Consequently, the reaction kinetics at the higher temperature catalyst surface are promoted while the reactions of lower temperature secondary gas-phase are reduced.

## Introduction

The environmental consequences due to the utilization of conventional oil resources and depletion of reserves have constrained the energy sector to pursue an alternative roadmap for the global demand outlook^1^. Presently, oil is a dominant energy vector, 33% of the global energy market, but the 1697.6 thousand million barrels discovered reserves will scarcely cover the energy demands for the subsequent 50 years^2,3^. Recently, natural gas has developed into the fastest growing energy and chemical production resource^4,5^ due to the widespread reserve availability, the development of new production methods and potential greenhouse gas benefits for the power sector. However, there are concerns with natural gas production from fugitive emissions, to potential land disturbance and water contamination of shale gas production to environmentally sensitive gas resources such as hydrates^6^.

Current prices and foreseen prices of natural gas are pushing producers to find more value through converting methane, the dominant constituent, to higher value chemicals. The prominent processes leading to the conversion of methane into syngas have been accentuated as a protuberant approach to preserve a carbon-neutral energy cycle in the prospective energy outlook^7^. Syngas, a gaseous mixture of hydrogen and carbon monoxide, is a valuable feedstock for multiple energy intensive industrial chemical processes^8^. Subsequently, numerous chemical processes have been pursued to transform methane into syngas, notably, steam reforming (SRM), partial oxidation (POx), and CO_2_ (dry) reforming processes (DRM). The DRM process has distinguished advantages due to the product composition ratio (H_2_/CO) and process flexibility^9,10^. Initially investigated by Fischer and Tropsch, the dry reforming of methane is an endothermic reaction producing a H_2_/CO ratio close to unity, providing a feedstock to chemicals production, methanol and long-chain hydrocarbons, through the Fischer-Tropsch process^11^. Moreover, the DRM reaction transforms two fundamental greenhouse gases, CO_2_ and CH_4_, providing a strong environmental advantage^12^. However, due to multiple thermodynamic equilibria, the DRM process is adversely impacted by the production of undesired by-products associated with the secondary gas-phase reactions: thermal degradation of methane, water gas shift reaction and carbon monoxide disproportionation^13^. These secondary gas-phase reactions drastically diminish the selectivity and quality of the syngas product. Many studies have concentrated on the development of a high performance catalytic system^14^. However, the industrial application of dry reforming has been hindered due to the lack of an effective and economical catalyst and the high energy requirements^15^. Various investigations on catalyst optimization and development have been reported in the literature to address the selectivity issue, however, there have been limited studies on the process heating approach^16^.

The exponential growth of renewable energy resources, namely, solar and wind power, provide a great opportunity to assist meet the demands of the energy market, and potentially help persevere the planet from further irrevocable destruction^17,18^. The recent record drops in the production and retail costs for solar energy due to advances in production and availability of feedstock has transformed the renewable energy generation from economically unfeasible to highly affordable^19^, providing clean and affordable electricity^20,21^. Therefore, the expediency of the affordable renewable electricity provides an astonishing prospect to perform chemical reactions by utilizing electromagnetic processes, namely, inductive, ultrasound and microwave heating.

Consequently, to address the productivity deficiency with the DRM process, this study has focused on the application of microwave heating for the gas-solid catalytic reactions. Selected advantages of microwave heating are: uniform, selective, and volumetric heating; high power density; instantaneous temperature control; reduced energy consumption; high reaction selectivity; less heat transfer limitations; process flexibility; and equipment portability. However, the essential feature of the microwave heating is regarded as selective heating. In general, exposure to microwave radiation increases the internal energy of the exposed material. The microwave interaction is correlated with the dielectric properties of materials associated with their physical and structural characteristics. However, many materials like gases, do not project substantial interaction with the microwave due to their inherent dielectric properties. Hence, solid microwave receptors, materials that dissipate the absorbed radiation to thermal energy effectively, have been developed. In contrary with conventional heating methods, the microwave heating approach to a gas-solid reactor creates a temperature gradient between the dielectric solid phase via the receptors and the gas phase. Hence, the higher local temperature in the solid phase promotes the catalytic reactions through higher reaction kinetics while the lower gas-phase temperature restricts the kinetics of the secondary gas-phase reactions. This study advocates that the application of the microwave heating approach can increase the productivity of the DRM process through improved selectivity of the syngas components, hydrogen and carbon monoxide, while maintaining a high conversion of the reactants, methane and carbon dioxide, simultaneously.

## Development of the Microwave Receptors

**Figure 1:**
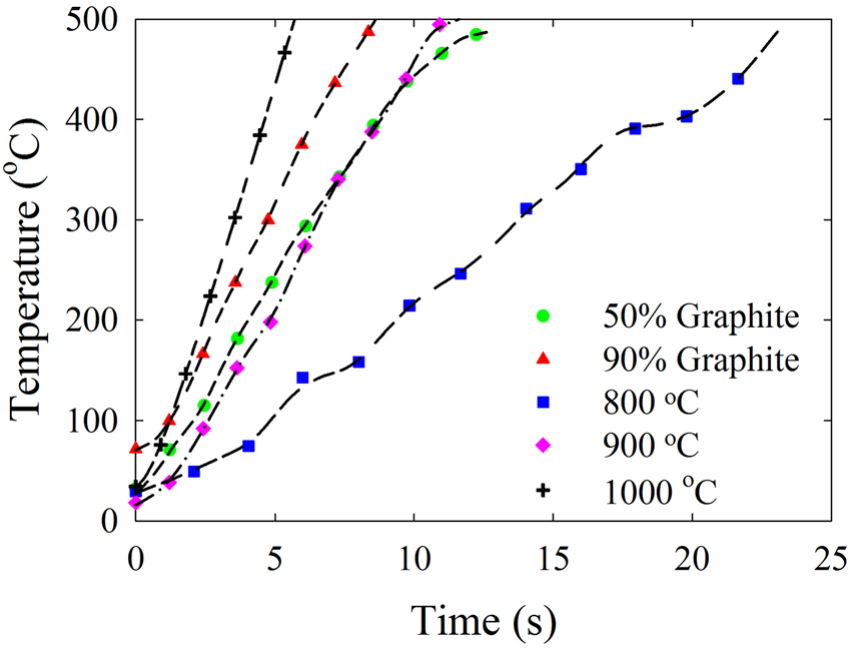
Comparative Microwave Heating Performance of 50% and 90% Graphite to Sand Mixtures and Coated Particles at 800, 900, 1000 °C and 240 Mins FBCVD Operational Conditions. The microwave heating performance of the C-SiO_2_ grades prepared at 800, 900 and 1000 °C and 240 minutes operating temperature and reaction times are compared with graphite and silica sand mixtures of 9:1 and 1:1. Although the C-SiO_2_ particles comprised of significantly lower carbon composition (Supplementary Table 1), they project superior microwave heating performance compared to the graphite/sand mixtures.

Due to the general deficiency of existing microwave receptor particles to generate a uniform temperature profile, along with the shortcomings of segregation, agglomeration and oxidation, a novel microwave receptor was developed. Chemical vapor deposition (CVD), assisted by induction-heating, of methane over silica sand particles was performed to develop C-SiO_2_ receptors, since carbon-coated receptors project superior properties as microwave receptors^22^. The developed C-SiO_2_ particles (particle density = 2650 kg/m^3^ and particle size = 212–250 *μ*m) fluid flow properties are classified as Geldart’s B which is advantageous for application in fixed and fluidized bed reactors. Thermogravimetric analysis and combustion infrared carbon detection (LECO) identified the carbon percentage of the samples prepared at the temperature range of 800 °C to 1000 °C and reaction time of 60- to 240- minutes to fluctuate in the range of 0.01 to 2.84 wt% (Supplementary Table 1). Scanning electron microscopy results demonstrated the effect of the operating conditions of carbon coating on the coated layer uniformity by diminishing the charging effect of the substrate silica sand particles (Extended Data Fig. 1). Furthermore, focused ionized beam (FIB) milling demonstrated the effect of the CVD temperature on the thickness of the carbon coating layer in the range of 19 ± 5 nm to 453 ± 16 nm, respectively (Extended Data Fig. 2). Furthermore, X-ray photoelectron spectroscopy (XPS) and energy dispersive X-ray spectroscopy (EDX) verified the effect of the CVD operating conditions on the composition of the coating layer and the formation of the electro-conductive graphite coating where the carbon constituent of the coating layer ranged from 1.2 to 95.4 atomic percentage (Extended Data Fig. 3, Supplementary Tables 2 and 3). In addition, the microwave heating performance of the developed C-SiO_2_ particles in the temperature range of 800 °C to 1000 °C and graphite-silica silica sand compositions of 50% and 90% were investigated (Fig. 1). The results demonstrated that although the carbon composition of the C-SiO_2_ was significantly lower, they generated a maximum heating rate of 100 °C/s massively exceeding the graphite-sand performance. Such a superior microwave interaction is associated with the generation of the electron freeways on the graphite network of the receptor particles^23^. Finally, the dielectric properties of the developed C-SiO_2_ were measured in a temperature range of 25 °C to 1000 °C (Extended Data Fig. 4), where the dielectric constant (*ε*′) is the ability of material to absorb electromagnetic wave, loss factor (*ε*′′) is the ability of material to dissipate the absorbed wave in the form of thermal energy and loss tangent (*tanδ*) is the ratio of loss factor to dielectric constant defining the efficiency of the microwave heating of the exposed material. The results for the dielectric constant, loss factor and loss tangent are 13.7, 6, 0.437, respectively, significantly exceed competitive conventional receptor particles^24^. The developed C-SiO_2_ particles when coated with the non-dielectric catalyst particles (non-absorbing of microwave) on the surface, is envisioned to generate high temperatures at the catalyst active sites as the receptor particles dissipate the heat outwards to the catalyst surface.

## Demonstration of the Gas and Solid Phase Temperature Profiles

**Figure 2:**
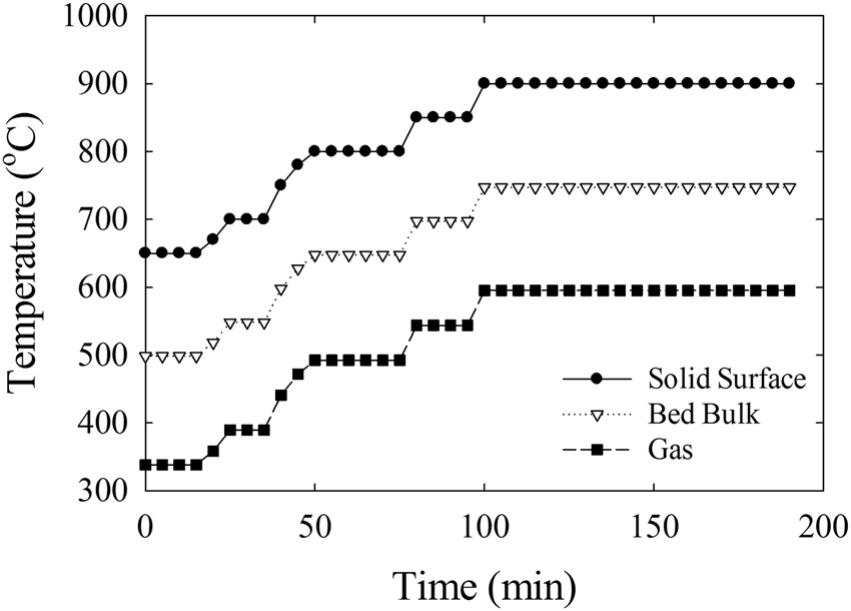
The Distribution of the Solid, Bulk and Gas Temperatures According to the DRM Operating Conditions in a Microwave-Assisted Fluidized Bed Reactor. The temperature profile of the solid surface and bulk associated with the reaction time was measured with radiometry and thermometry methods. The significant gradient between the two temperature profiles is evident. However, due to the complex status, the gas temperature profile was estimated using energy balance equations and empirical equations (see citation 25). The results confirm the selective microwave heating mechanism and the opportunity to optimize gas-solid catalytic reactions.

The microwave selective heating mechanism leverages materials with substantial dielectric properties to interact with microwaves leading to heat generation, while dielectric properties of gases and the reactor material (Supplementary Table 4) have minimal interactions. This leads to a temperature gradient between the solid phase (C-SiO_2_ receptors and HiFUEL R110 catalyst particles mixture) and the gas phase (nitrogen). The temperature measurement of the solid surface and bulk, a contributive state of the solid and gas phases, using radiometry and thermometry methods, respectively, illustrates an astounding gradient across the superficial gas velocities (Extended Data Figs 5 and 6). The measured temperature gradient demonstrates the anticipated lower temperature of the gas phase compared to the solid particles. However, due to the physical and chemical structure, the direct measurement of the gas phase temperature was not possible. Thus, the gas phase temperature was estimated with the assistance of energy balance equations and empirical correlations^25^. The experimental data and the estimated correlations results demonstrated a remarkable temperature gradient between the gas and the solid phase at the DRM reaction conditions (Fig. 2). These measurements reaffirm the hypothesis that the productivity of the catalytic gas-solid reactions is ptentially enhanced due to the localized temperature of the catalyst surface being significantly higher than the gas phase.

## Dry Reforming of Methane Optimization

Dry reforming of methane is an endothermic reaction, expressed as:

1 $$C{H}_{4}+C{O}_{2}\to 2CO+2{H}_{2}\,{\rm{\Delta }}{H}_{298}^{0}=+\,247\,kJ\,mo{l}^{-1}$$

Moreover, the resilient C-H bond necessitates the application of an appropriate catalyst to initiate the reaction. Although signified as a renowned process to produce syngas, the complex reaction pathway deteriorates the quality of the final product due to secondary gas-phase reactions (Supplementary Table 6). Furthermore, the production of carbon residues accelerates the deactivation of the catalyst active sites, whereas thermal degradation of methane,

2 $$C{H}_{4}\to C+2{H}_{2}\,{\rm{\Delta }}{H}_{298}^{0}=74.9\,kJ\,mo{l}^{-1}$$

water gas shift reaction,

3 $$CO+{H}_{2}O\leftrightarrow C{O}_{2}+{H}_{2}\,{\rm{\Delta }}{H}_{298}^{0}=-\,41.2\,kJ\,mo{l}^{-1}$$

and carbon monoxide disproportionation (Boudouard reaction)

4 $$2CO\leftrightarrow C+C{O}_{2}\,{\rm{\Delta }}{H}_{298}^{0}=-\,172.4\,kJ\,mo{l}^{-1}$$

have been profoundly restricting the DRM reaction productivity. The application of transition metal catalysts, namely, nickel, has been studied for the DRM process due to the high reactivity with methane and the potential lower economic costs^16,26–28^. However, the vulnerability of the nickel-based catalysts to carbon deposition has been thermodynamically inevitable^29^. Hence, HiFUEL R110 nickel based (15–20 wt% Ni, Alfa Aesar) alumina supported particles were selected as the catalytic system mixed with the C-SiO_2_ receptor particles. The DRM reactions were performed at an equivalent ratio of unity (CO_2_/CH_4_=1:1), to emphasise the effect of the microwave heating mechanism on the process results.

**Figure 3:**
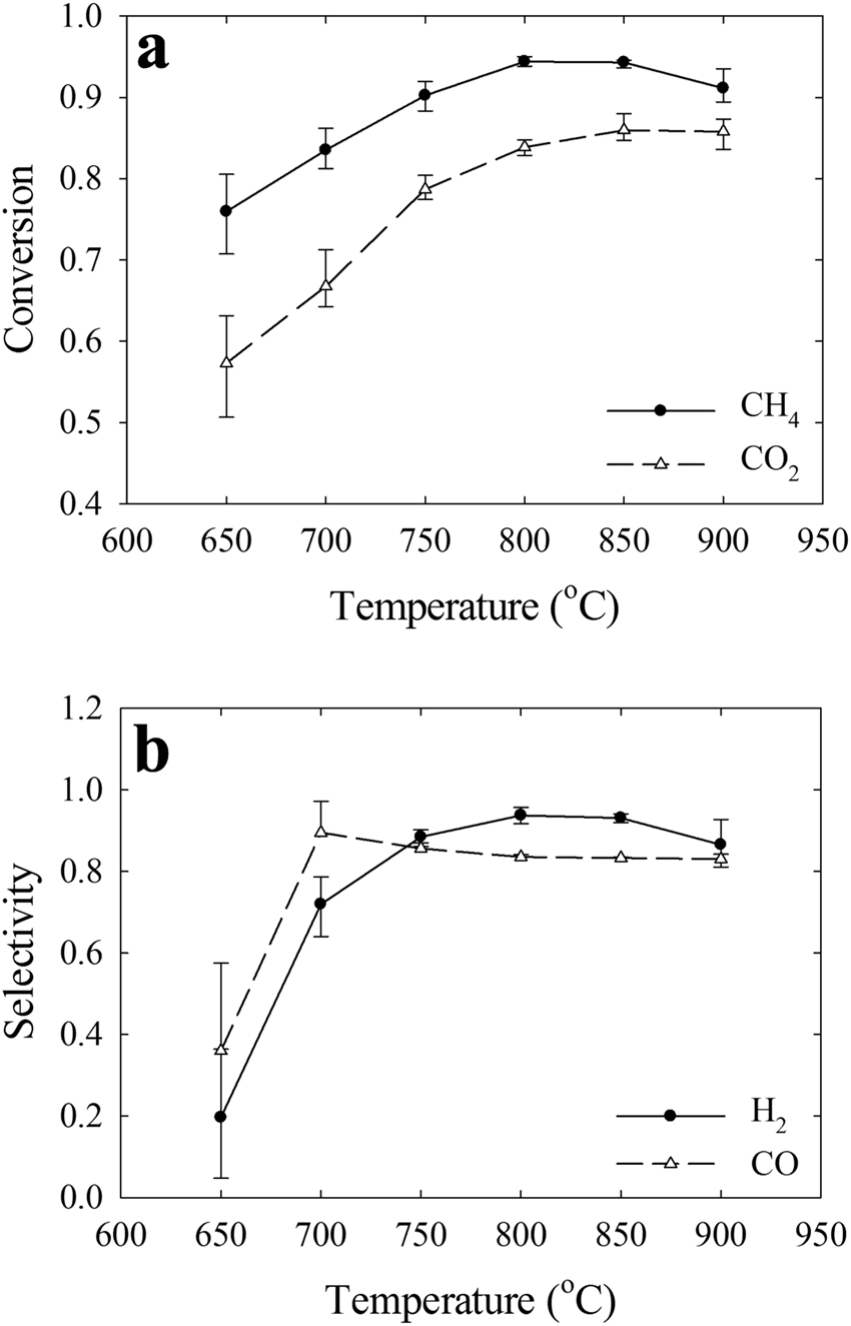
The Evolution of the Reactants and Syngas Components According to the DRM Operating Conditions. (**a**) The conversion of the DRM reactants CH_4_ and CO_2_ is enhanced by increasing the reaction temperature, which is in compliance with the endothermic nature of the reactions. Although the conversion of CH_4_ is superior to carbon dioxide since CO_2_ is the limiting reactant at CO_2_/CH_4_ < 1. (**b**) The syngas components, H_2_ and CO, demonstrate an increasing trend for selectivity at higher operating temperatures. However, CO selectivity is maximum at 650 °C due to the dominance of the reverse CO disproportionation reaction at lower temperatures.

The conversion of the reactants, CH_4_ and CO_2_, was observed across the DRM operating temperature from 650 °C to 900 °C (Fig. 3a). The results were in compliance with the endothermic nature of the DRM process^30–33^. The CH_4_ conversion ranged from 80% to a threshold of 95% and the CO_2_ equivalent conversion from >60% to >85%. Such difference in the conversion of the reactants is due to the methane decomposition reaction and formation of carbon, even though at CO_2_/CH_4_ ratios of 0.5 to 1, CO_2_ is typically the limiting reactant^30^. Meanwhile, the selectivity of H_2_ was enhanced up to 95% by increasing the DRM operating temperature prior to the catalyst deactivation (Fig. 3b). Moreover, at CO_2_/CH_4_ ratios of close to unity, the H_2_ selectivity is further improved due to the persistent decomposition of methane. However, increasing the operating temperature diminished the H_2_ selectivity enhancement due to the reverse water gas shift reaction^34,35^. Moreover, the deactivation of the catalyst intensified at higher operating conditions contributed to a decline in the H_2_ selectivity^36^. In contrast, the CO selectivity reached a maximum value close to 100% at 700 °C, but due to the formation of carbonaceous material on the receptor particles, the CO disproportionation reaction was enhanced as temperature increased. Consequently, due to the limitations of CO_2_ reactions at CO_2_/CH_4_ ratios close to unity, the CO selectivity further encountered a distinctive decline period. However, at higher temperatures above 800 °C, the CO selectivity recovered slightly due to the endothermic mechanism of the originating reactions. In general, the conversion of the reactants and the selectivity of the products exceeded the estimated thermodynamically predicted equilibrium values and the available studies in the literature^14^. The exceptional productivity of the DRM process is associated with the microwave selective heating mechanism, which significantly restricts the evolution of the secondary gas-phase reactions while maintaining a high conversion of the reactants and high selectivity of the syngas components.

**Figure 4:**
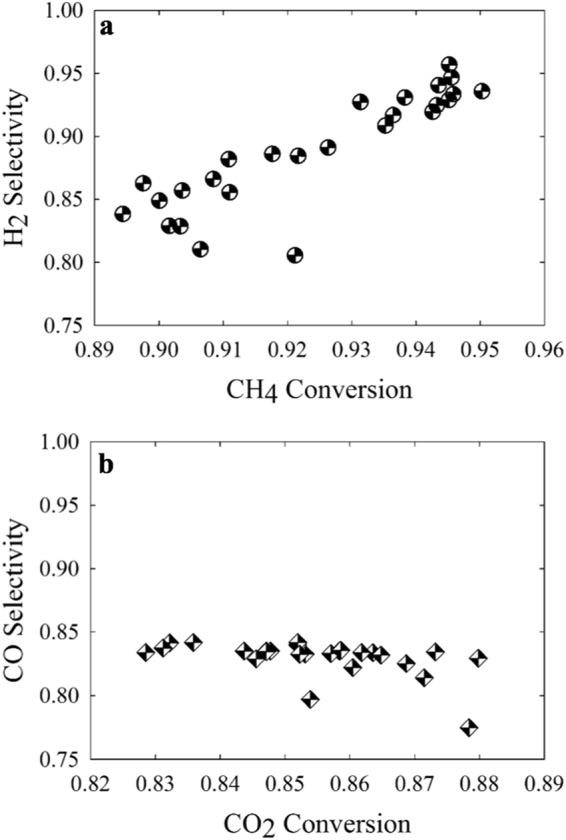
Distribution of the Selectivity of the Syngas Products According to the Conversion of the Reactants. (**a**) The selectivity of H_2_ is proportional to the conversion of CH_4_, where both conversion and selectivity maintain high values, simultaneously. The observations highlight that the microwave selective heating approach diminishes secondary gas phase reactions. (**b**) The selectivity of CO is relatively flat with increased conversion of CO_2_ maintaining high conversion and selectivity. The observations highlight that the microwave selective heating approach diminishes secondary gas phase reactions.

In general, while performing catalytic gas-solid reactions, the conversion of the reactants and the selectivity of the desired product are respectively inversely proportional due to the evolution of the secondary gas-phase reactions^16^. However, the microwave dry reforming of methane maintained a high conversion of the reactants and selectivity of the syngas components at an operating temperature range of 800 °C to 900 °C (Fig. 4). The results established a superior productivity over the equivalent conventional process heating studies in the literature which required to compromise between the conversion and selectivity values^37–39^. The simultaneous enhanced conversion and selectivity were the distinctive achievement of the microwave heating mechanism^40–42^. Thus, the application of the microwave heating mechanism is gaining commercial interest due to the reduced energy requirements, improved potential economics of the reforming reactions and quality of the final product.

## Conclusions

The application of natural gas for energy and chemicals production has an unprecedented influence on the global environmental and economic outlook. However, sustained low prices and challenges associated with the accessibility, transportation and distribution of the natural gas necessitates the development of an effective conversion method to value-added products. Dry reforming of methane has been highlighted as a conversion process with potential environmental benefits, process flexibility and economic value. With cheap and abundant renewable energy leveraging electricity for chemical processes has become attractive. Leveraging process heating approaches such as microwave, induction and ultrasound may enable this renewable electricity to be further valorized. Leveraging the inherent nature of materials interaction with microwave, the approach taken in this work was able to create strong thermal gradients between the solids and the gas in a gas-solid reactor. The microwave process helped promote the selectivity of the desired products and maintain the conversion of the reactants simultaneously while reducing the kinetics of the secondary gas phase reactions (Extended Data Fig. 7).

A novel C-SiO_2_ microwave receptor was developed by chemical vapor deposition of methane over silica sand substrate. The resultant C-SiO_2_ particles had a high coating uniformity and a high microwave interaction. This permitted for a uniform heating of the particles under microwave irradiation and the resultant particles flow properties made them amenable to be used in a fluidized or fixed bed. A future direction of development of microwave receptors is particles with higher specific surface area to accommodate catalyst active sites.

The measurement of the gas and solid phase temperature profiles with the assistance of correlations and direct measurement techniques demonstrated strong temperature gradients (~300 °C) are possible. But the development of direct gas temperature measurement techniques is essential to monitor the gas-phase temperature profile within the reactor to further optimize the operating conditions.

The DRM reaction results emphasize the significance of the microwave heating mechanism on the productivity of gas-solid catalytic reactions. The effect of microwave processing of the chemical reactions has been recurrently presented in the literature^41,43–46^. The conversion of the reactants, CH_4_ and CO_2_ and selectivity of the syngas components, H_2_ and CO, employing a basic nickel based catalyst demonstrated an exceptional outcome comparable to advanced catalyst systems^47^.

For the future, the application of microwave heating may diminish the energy requirements of the processes since the heat generation is concentrated through the dielectric material. Furthermore, the environmental benefits of the microwave-heated reactions associated with the renewable electricity resources are encouraging. In addition, the effect of microwave heating on the kinetic parameters, activation energy and pre-exponential factor, demonstrates valuable information to understand the mechanism of the catalytic reactions and formation of the intermediate components, correspondingly^48–50^. Furthermore, microwave heating mechanism is recommended as a prospective technique to clarify reactions mechanisms by distinguishing between the catalytic and gas-phase reactions. Ultimately, the deposition of the catalyst components on the surface of the receptor particles, thus coupling the catalyst and receptor material into a unified system, would possibly enhance the effect of the microwave heating process, while minimizing segregation and agglomeration that plague gas-solid systems.

## Electronic supplementary material

Supplementary Information
