# Supplementary material for: Microwave Heating-Assisted Catalytic Dry Reforming of Methane to Syngas
Source: Sci Rep. 2018 Jun 12;8:8940. doi: 10.1038/s41598-018-27381-6 (PMC5997706; doi:10.1038/s41598-018-27381-6)
Supplement: Supplementary file 1 — Supplementary Information [file 41598_2018_27381_MOESM1_ESM.pdf]

Supplementary Information

Microwave Heating-Assisted Catalytic Dry Reforming of Methane to Syngas

Sepehr Hamzehlouia[1], Shaffiq A. Jaffer[2] and Jamal Chaouki[1*]

[1]Department of Chemical Engineering, Polytechnique Montreal, c.p. 6079, Succ. Centre-ville, Montreal, Quebec, H3C 3A7, Canada

[2]Total American Services, Inc., 82 South St., Hopkinton, MA, 01748

*Correspondence and requests for materials should be addressed to jamal.chaouki@polymtl.ca

Methods

Development of the C-SiO$_2$ receptors. The receptor particles were developed by induction heating-assisted chemical vapor (CVD) deposition of methane (99.92%) over silica sand substrates (ρ_p= 2.6 g/cm^3, d_p= 212- 250 μm)[51]. A 10 KW power source provided the high frequency and voltage electrical field for the induction heating applications. The CVD reactions were performed in a in a 2.5-cm OD, 0.3-cm width and 30-cm long tubular reactor (stainless steel grade 316 tubular). A water-cooled brass/copper alloy coil transferred the electrical field to the workpiece (Extended Data Fig. 8). For each experiment 60 g of the silica sand was loaded to the reactor, and the particles bed was fluidized with nitrogen (99.99%) in a bubbling regime. The experiments were performed at 800, 900 and 1000°C operating temperatures and 60-, 120- and 240 -minute reaction times to investigate the effect of the reaction parameters on the evolution of the coating layer. The superficial gas velocity was adjusted during the experiments according to the operating temperature in a range of 3.3 cm/s to 10 cm/s to maintain the bubbling fluidization regime.

Carbon layer characterization. The carbon composition of the C-SiO$_2$ particles developed in various operating conditions was assessed by a TA Instruments TGA Q 5000 thermogravimetric analysis (TGA) device. The measurements were performed in a temperature range of 25 to 1000°C and at a heating rate of 10°C/min under air atmosphere with a flow rate of 20 mL/min and Nitrogen as the purge gas at 20 mL/min. The results were further verified by combustion infrared carbon detection technique deploying a LECO CS744 series carbon analyzer with a LECOCEL II and an iron chip accelerator (Supplementary Table 1). The accelerator and sample temperature were adjusted at 1800°C and 1400°C, respectively. Detailed analyses on the morphologies of the samples prepared at various operating conditions were conducted by field emission scanning electron microscopy using SEM-FEG; model JSM-7600 TFE, JEOL, Japan (Extended Data Fig.

1). The SEM was performed in LEI imaging mod, operating distance of 15 mm and 5 kV. Furthermore, focused ionized beam (FIB) milling (FIB; model 2000-A, Hitachi, Japan) was deployed to measure the carbon coating layer, whereas the samples were initially cavum coated with tungsten and later milled through in a rectangular area of 30x10 μm^2 at 20 kV. The milled samples were further investigated with the SEM-FEG, and the thickness of the coating layers was determined using the open source Image-J software (Extended Data Fig. 2). The composition of the coating layer was investigated by energy-dispersive X-ray spectroscopy (EDX; Oxford Instruments), and the results were investigated by the corresponding software (Extended Data Fig. 3 and Supplementary Table 2). Finally, the surface characterization of the coating layer was accomplished by X-ray photoelectron spectroscopic (XPS) analysis carried out on a VG scientific ESCALAB 3 MK II X-ray photoelectron spectrometer using a MG Kα source (15 kV, 20 mA). Each scan was performed at pass energy of 100 eV and energy step size of 1.0 eV at 10-nanometer penetration depth (Supplementary Table 3).

MW heating performance. The microwave heating performance of the developed C-SiO_2 receptor particles was executed in a single-mode microwave heating-assisted lab-scale fluidized bed reactor. The results were further compared with 50% and 90% graphite/sand samples (Fig. 1). A water-cooled 2.5 kW, 2.45 GHz Genesys Systems microwave generator was employed to transmit the microwave radiation. The microwave radiation was directed to a 2.5 cm OD and 8 cm length tubular quartz reactor (Extended Data Figure 9). For each experiment, 30 gr of the sample was loaded to the quartz reactor, and the temperature was monitored with a K type thermocouple. The receptor bed was fluidized with nitrogen in a bubbling regime and maintained by adjusting the superficial gas velocity through the experiments. The samples were heated up to 500^oC to determine the corresponding heating rate.

Temperature profile distribution.

A single-mode microwave heating-assisted lab-scale fluidized bed reactor was developed. Microwave radiation was generated by a 2.5 KW and 2.45 GHz frequency water-cooled Genesys system magnetron and transferred to a 20-cm height and 2.24-cm ID quartz tube reactor (Extended Data Fig. 10). For each experiment, 30 g of the receptor samples were loaded to the reactor. The experiments were performed in 3.4 cm/s, 6.7 cm/s and 10 cm/s and 500, 600 and 700°C superficial gas velocities and particle surface temperatures, respectively (Extended Data Figs. 5 and 6). The temperature measurement of the dielectric solid particles (C-SiO_2) and bed bulk was performed using radiometry and thermometry methods, respectively, whereas a thermopile was calibrated to convert the voltage generated from the light capturing of the particles radiation exposed to microwave heating to a corresponding temperature. Due to the complex measurement of the gaseous components exclusively, the gas phase temperature was estimated with energy balance equation and empirical correlations developed by the attained experimental data (Fig. 2)[25].

MW-assisted DRM. The DRM reactions were attained in a lab-scale single-mode microwave-heated fluidized bed reactor. The microwave radiation was generated by a 2.5 kW and 2.45 GHz frequency Genesys system magnetron with an internal water cooling mechanism and transferred to a 2.54-cm ID and 20-cm long fused quartz tube reactor (Extended Data Fig. 11). For each experiment, a mixture of 12 gr of the HiFUEL R110 (15-10% Ni, alumina supported) and 28 gr of the C-SiO_2 receptor/promoter were loaded to the quartz reactor. The bed was fluidized by nitrogen with a superficial gas velocity of 3.3 cm/s to 10 cm/s depending on the system temperature to maintain a bubbling fluidization regime. The temperature of the solid particles was monitored with a thermopile. Due to the insufficient dielectric properties of the system components and the bed constituents, the C-SiO2 receptors mitigated for the heat generation inside the reactor, exclusively.

The gas flow was switched to a CO_2/CH_4=1:1 following the accomplishment of the operating temperature. The exhaust gas was continuously monitored by a Varian CP-4900 micro gas chromatographer (GC) to detect the volumetric fraction of each component. The reactions were terminated upon the complete deactivation of the catalyst active sites due to the extended carbon deposition (Extended Data Fig. 12).

Supplementary Table 1 - TGA and Combustion Infrared Carbon Detection (LECO) Results for the Original and Coated Particles at Various Coating Times and Temperatures

Reaction Temperature (°C)	Pure Sand	800			900			1000		
Reaction Time (min)	N/A	60	120	240	60	120	240	60	120	240
TGA Carbon (wt%)	0.02	0.04	0.05	0.06	0.05	0.09	0.27	0.31	1.90	2.84
LECO Carbon (wt%)	<0.01	<0.01	<0.01	0.02	0.01	0.1	0.25	0.27	1.83	2.75

The carbon composition of the C-SiO_2 samples prepared at various operating conditions was determined by TGA and verified with LECO.

Supplementary Table 2 - Spectrum Analysis of EDX Data According to Extended Figure 3 Acquisitions

Composition (%)	Spectrum Number Based on Extended Data Figure 3																
	1	2	3	4	5	6	7	8	9	10	11	12	13	14	15	16	17
C	1.6	1.3	1.2	1.6	8.2	3.1	24.1	91	93.9	87.4	95.3	94.3	95.4	91.8	89.7	89.8	89.6
Si	37.7	44.6	42.5	35.8	48.1	83.7	11.5	1.1	2.6	7.2	2.6	2.4	3.3	2.4	3.4	3.8	4.8
O	50	48.4	52.7	50.3	7.9	6.7	7	1	3.5	5.4	2.1	3.2	1	4.9	5.2	5.8	4.9

The carbon, silicon and oxygen composition of the carbon coating layer for multiple receptor grades were investigated by EDX (Extended Data Fig. 3) to determine the uniformity of the coating layer accordingly.

Supplementary Table 3 - XPS Data Analysis for Original and Coated Particles at Various Coating Times and Temperatures

Element	B.E.	S.F.	Pure Sand	800			900			1000		
			N/A	60	120	240	60	120	240	60	120	240
			Relative Atomic Percentage (%)									
AL2p	74.4	0.185	5.3	6.9	6.0	5.7	4.7	1.3	0.3	6.2	0.7	0.7
Si2p	102.9	0.270	17.7	16.8	15.7	15.0	11.0	4.1	1.2	13.7	2.3	1.7
C1s	284.9	0.250	7.6	16.5	25.1	27.3	50.6	78.6	92.9	27.5	89.9	91.4
Ca2p	348.1	1.580	0.6	0.5	0.2	0.1	---	---	---	0.4	---	---
K2s	377.8	0.387	1.4	1.9	1.5	2.0	0.9	---	---	2.0	---	---
N1s	401.7	0.420	0.8	---	---	---	---	---	---	---	---	---
O1s	532.0	0.660	64.3	57.4	50.7	50.0	32.2	15.6	5.5	48.7	6.9	6.2
F1s	688.6	1.000	1.4	---	0.9	---	0.6	---	---	1.5	0.3	0.4
Na1s	1071.6	2.300	0.8	---	---	---	---	---	---	---	---	---

The XPS results reflected the quality of the uniformity and composition of the coated carbon layer according to the CVD operating conditions.

Supplementary Table 4 - Dielectric Properties of the Employed Material at Ambient Temperature and 2.45 GHz Frequency

Material	Dielectric Constant (ε')	Loss Factor (ε'')	$tan\delta$
Silica Sand	3.066 [52]	0.215 [52]	0.070 [52]
Carbon	7 [53]	2 [53]	0.285
C-SiO_2	13.7	6	0.437
Nitrogen	1.00058 [54]	-	-
Fused Quartz	4.0 [55]	0.001[55]	0.00025

The dielectric properties of the reactor and bed material verified that C-SiO_2 particles are exclusively responsible for the heat generation of the process. The dielectric properties of the receptors are according to the measurements reported in the present study (Extended Data Fig. 4)

Supplementary Table 5 - Complete Reaction Mechanism Pathways for Dry Reforming of Methane [30]

Reaction #	Reaction	ΔH_{298}(kJ/mol)
1	$CH_4 + CO \leftrightarrow CO + 2H_2$	247
2	$CO_2 + H_2 \leftrightarrow CO + H_2O$	41
3	$2CH_4 + CO_2 \leftrightarrow C_2H_6 + CO + H_2O$	106
4	$2CH_4 + 2CO_2 \leftrightarrow C_2H_4 + 2CO + 2H_2O$	284
5	$C_2H_6 \leftrightarrow CH_4 + H_2$	136
6	$CO + 2H_2 \leftrightarrow CH_3OH$	-90.6
7	$CO_2 + 3H_3 \leftrightarrow CH_3OH + H_2O$	-49.1
8	$CH_4 \rightarrow C + 2H_2$	74.9
9	$2CO \rightarrow C + CO_2$	-172.4
10	$CO_2 + 2H_2 \leftrightarrow C + 2H_2O$	-90
11	$H_2 + CO \leftrightarrow H_2O + C$	-131.3
12	$CH_3OCH_3 + CO_2 \leftrightarrow 3CO + 3H_2$	248.4
13	$3H_2O + CH_3OCH_3 \leftrightarrow 2CO_2 + 2H_2$	136
14	$CH_3OCH_3 + H_2O \leftrightarrow 2CO + 4H_2$	204.8
15	$2CH_3OH \leftrightarrow CH_3OCH_3 + H_2O$	-37
16	$CO_2 + 4H_2 \leftrightarrow CH_4 + 2H_2O$	-165
17	$CO + 3H_2 \leftrightarrow CH_4 + H_2O$	-206.2

The possible DRM reactions pathways according to the thermodynamic studies. Reaction (1) is the anticipated root to produce syngas, while reactions (2) to (17) deteriorate the quality of the final product by evolution of the secondary by-products or deactivation of the catalyst active sites through carbon deposition.

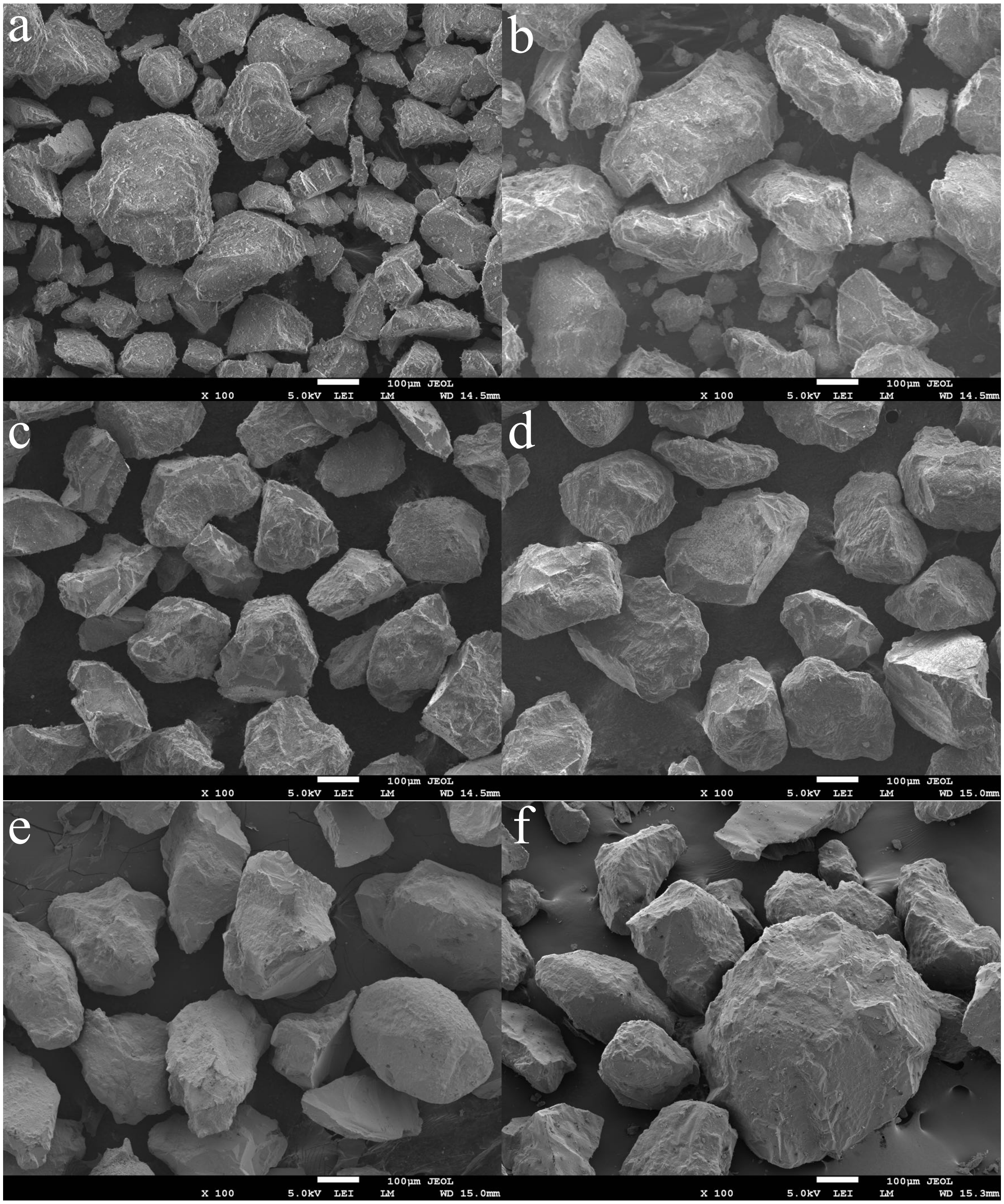

Extended Data Figure 1 | Representative SEM Observation of the Developed C-SiO_2 Particles. **a**, pure sand projected extensive charging effect under the emission. **b**, coated sand at 800°C and 60 mins reduced the charging effect, although the core silica particles are evidently visible. **c**, coated sand at 800°C and 120 mins increased the quality of the coating although traces of the core particle are still visible. **d**, coated sand at 900°C and 60 mins considerably restricted the charging effect. **e**, coated sand at 900°C and 240 mins diminished the charging effect completely due to the uniform coating of the particles. **f**, coated sand at 1000°C and 240 mins continued the trend to enhance the uniformity of the coating layer.

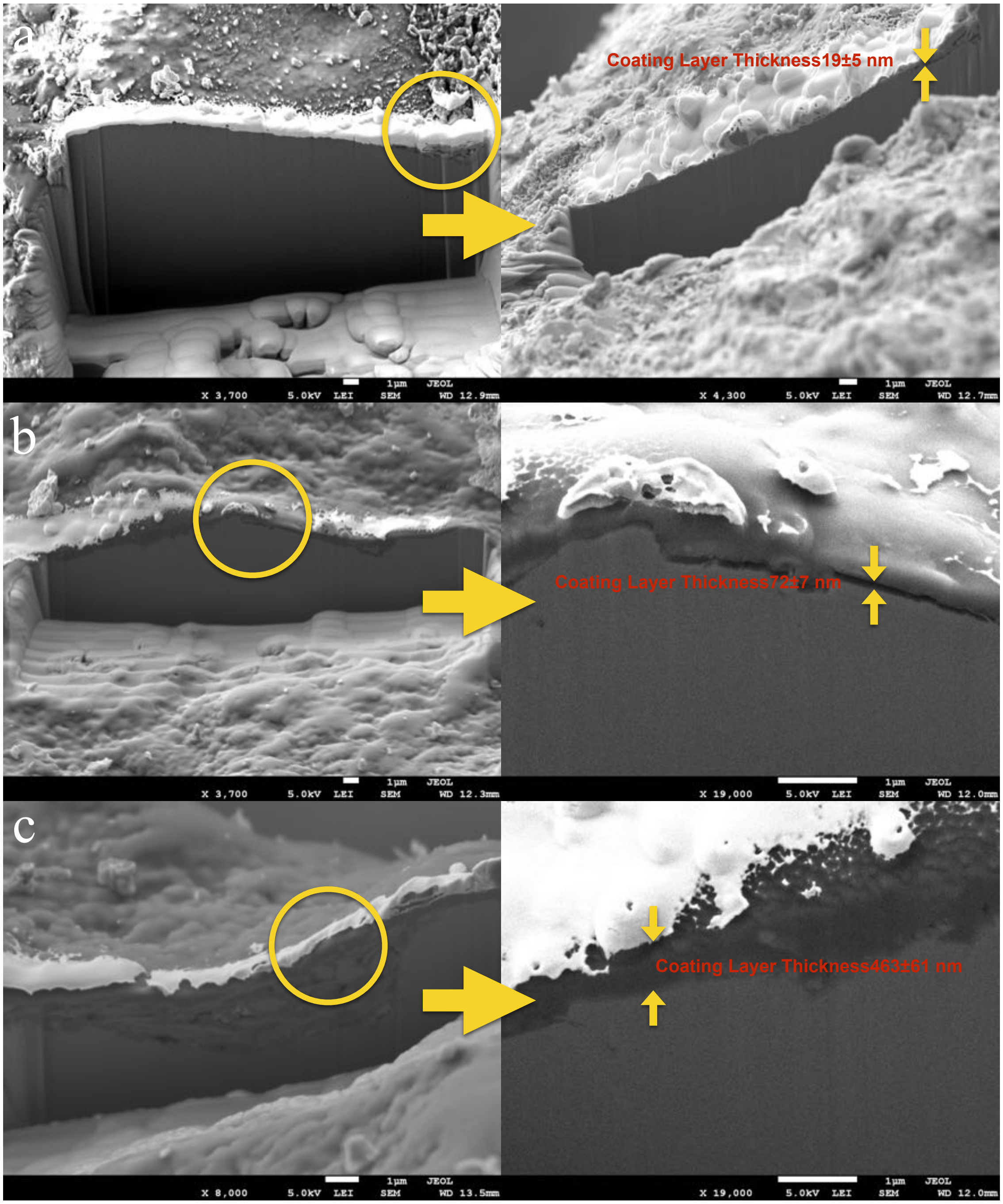

Extended Data Figure 2 | Representative SEM Images of the Evolution of the Coating Layer Thickness Using FIB Milling. a, The coating layer thickness for particles prepared at 800ºC and 240 minutes CVD temperature and time is 19±5 nm, which advocates the insignificant operating conditions. **b**, The coating layer thickness for particles prepared at 900ºC and 240 minutes CVD temperature and time is 72±7 nm, which advocates operating conditions have improved. **c**, The coating layer thickness for particles prepared at 1000ºC and 240 minutes CVD temperature and time is 463±61 nm, which advocates the operating conditions have been enormously amended.

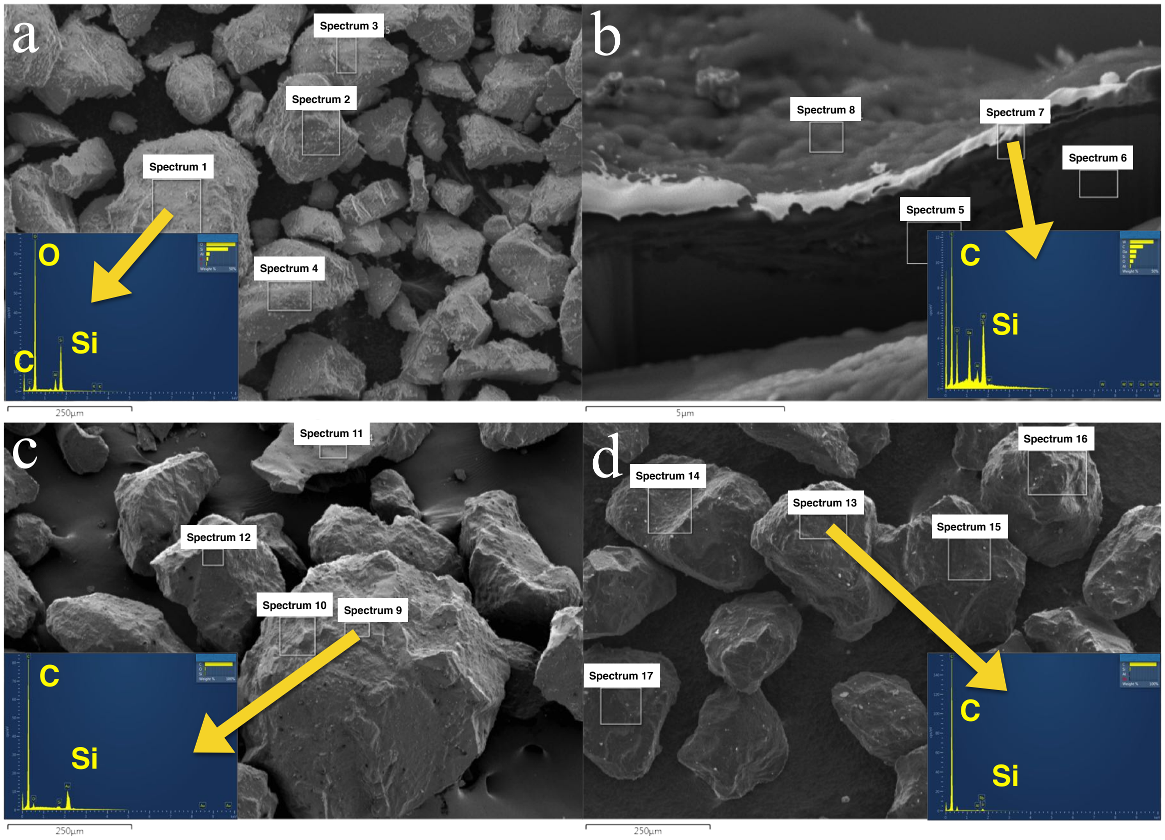

Extended Data Figure 3 | Selective EDX Results to Verify the Uniformity and Composition of the Coating Layer a, uncoated sand particles scan developed a reference baseline to evaluate the uniformity of the carbon coating layer on the C-SiO_2 particles. **b**, 800°C and 240 mins coated particles projected higher uniformity since the carbon spectrum was enhanced; however, the oxygen and silica spectrums are yet visible. **c**, 900°C and 240 mins coated particles further enhanced the uniformity of the coating layer although traces of the core particles are yet visible. **d**, 1000°C and 120 mins coated particles projected exceptional uniformity of the coating layer, where the oxygen and silicon peaks were diminished.

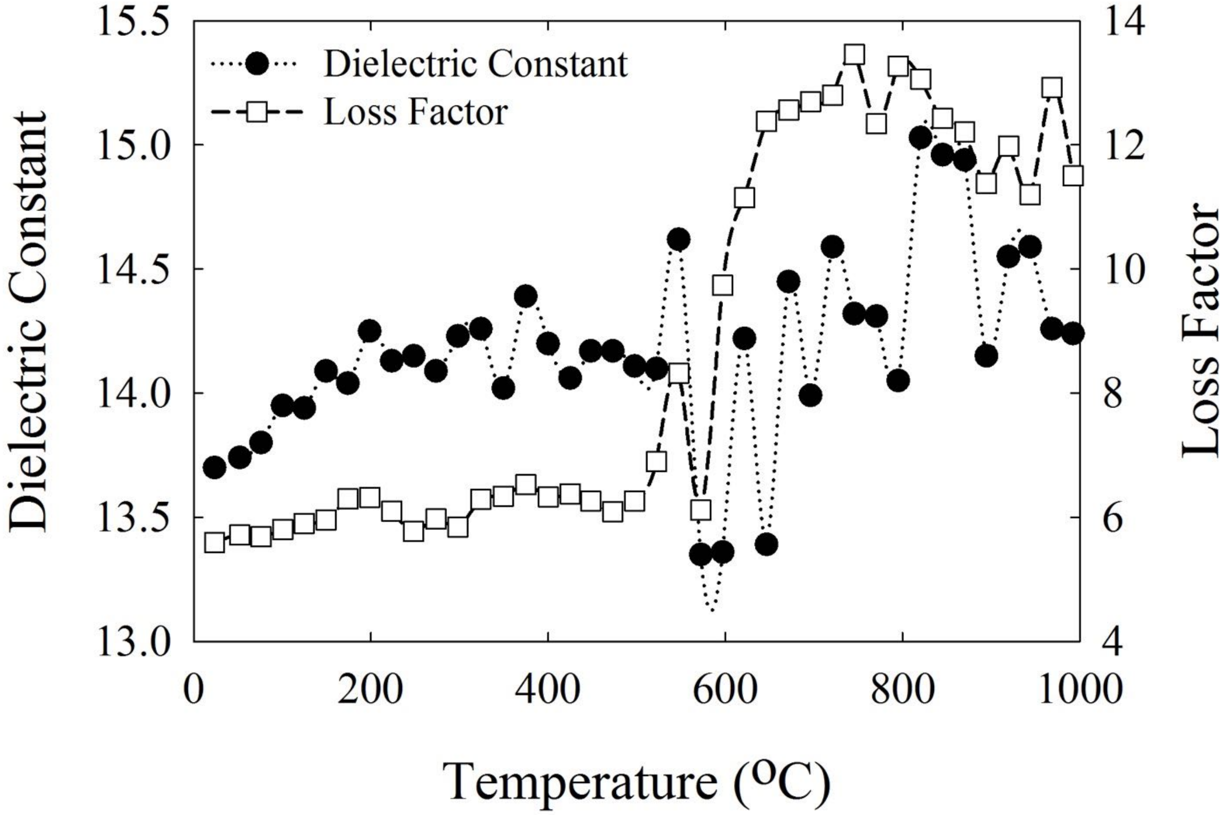

Extended Data Figure 4 | Effect of the Operating Temperature on the Dielectric Constant and the Loss Factor of the Developed C-SiO_2 Particles. The dielectric properties are a function of operating temperature frequency, whereas the frequency value is stationary at 2.45 GHz for microwave heating purposes. The dielectric constant, the ability of the dielectric material to absorb microwave radiation, does not project significant fluctuation while enhancing the operating temperature. However, the loss factor, the ability to dissipate the absorbed microwave radiation to heat, is enormously manipulated by the operating temperature, whereas the loss factor was enhanced by 100% in a temperature range of 25°C to 1000°C. It implies that the efficiency of the receptors could be doubled during the reaction period, hence enhancing the effect of the selective microwave heating mechanism.

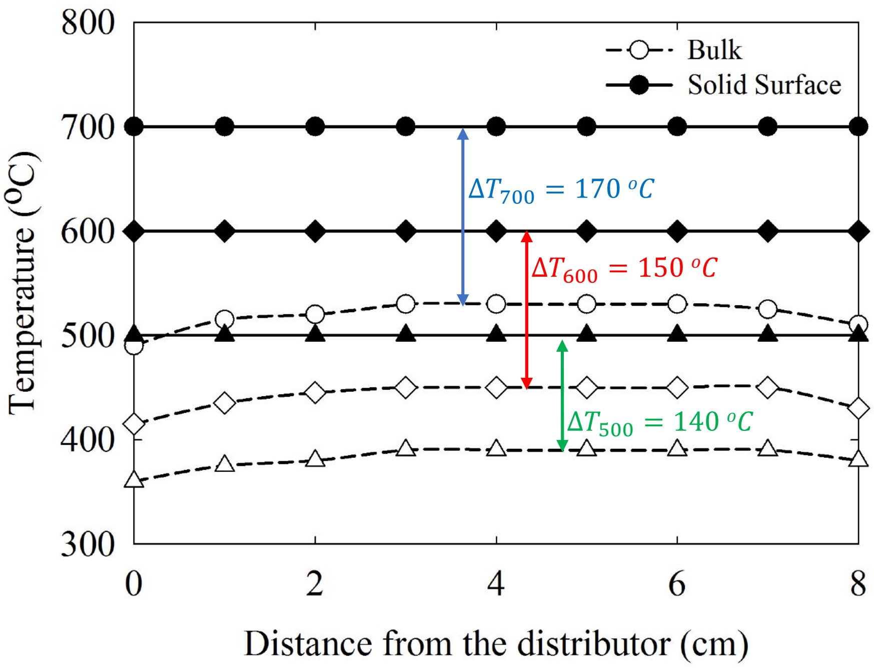

Extended Data Figure 5 | Effect of the Operating Temperature on the Solids and Bulk Temperature in the C-SiO$_2$ Receptor Bed at $\boldsymbol{U_g}$= 6.7 cm/s. The particle surface temperature affects the solid phase and the bed bulk temperature gradient. Increasing the solid surface temperature causes the gradient value to expand, while the effect is similar to the superficial gas velocity (See Extended Data Fig 6.). Thus, operating at higher temperatures demonstrates a more prominent opportunity to advocate the effect of the microwave selective heating mechanism due to the evident larger temperature gradient.

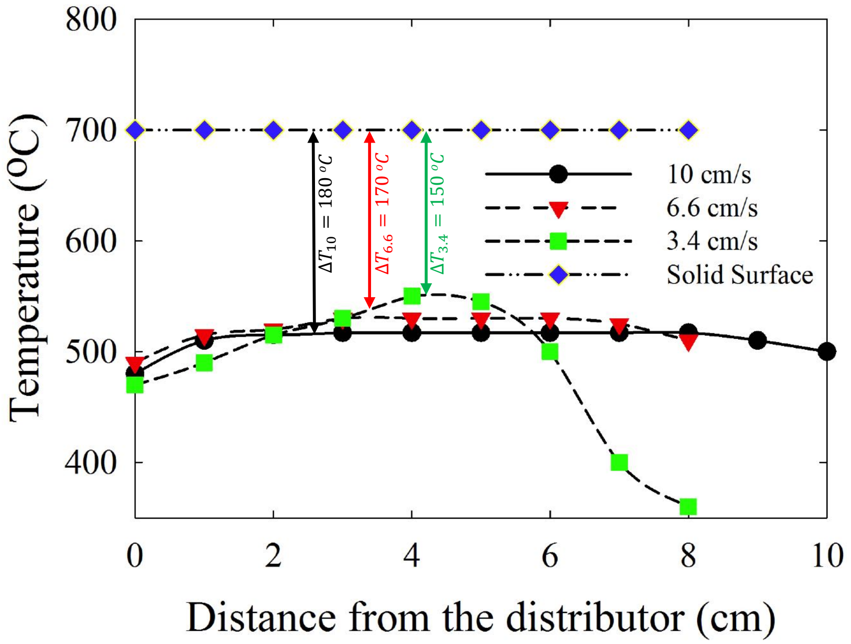

Extended Data Figure 6 | Effect of Superficial Gas Velocity on the Solids and Bulk Temperature Distribution in the C-SiO_2 Receptor Bed at Solid Surface Temperature of 700°C. Superficial gas velocity dominantly influences the temperature gradient between the gas and solid phase. Due to the inability of the gases to adapt to the reactor temperature at lower residence times, the gradient expands by increasing the superficial gas velocity. Consequently, the application of higher superficial gas velocities demonstrated the effect of microwave selective heating mechanism on the productivity of the reactions extensively.

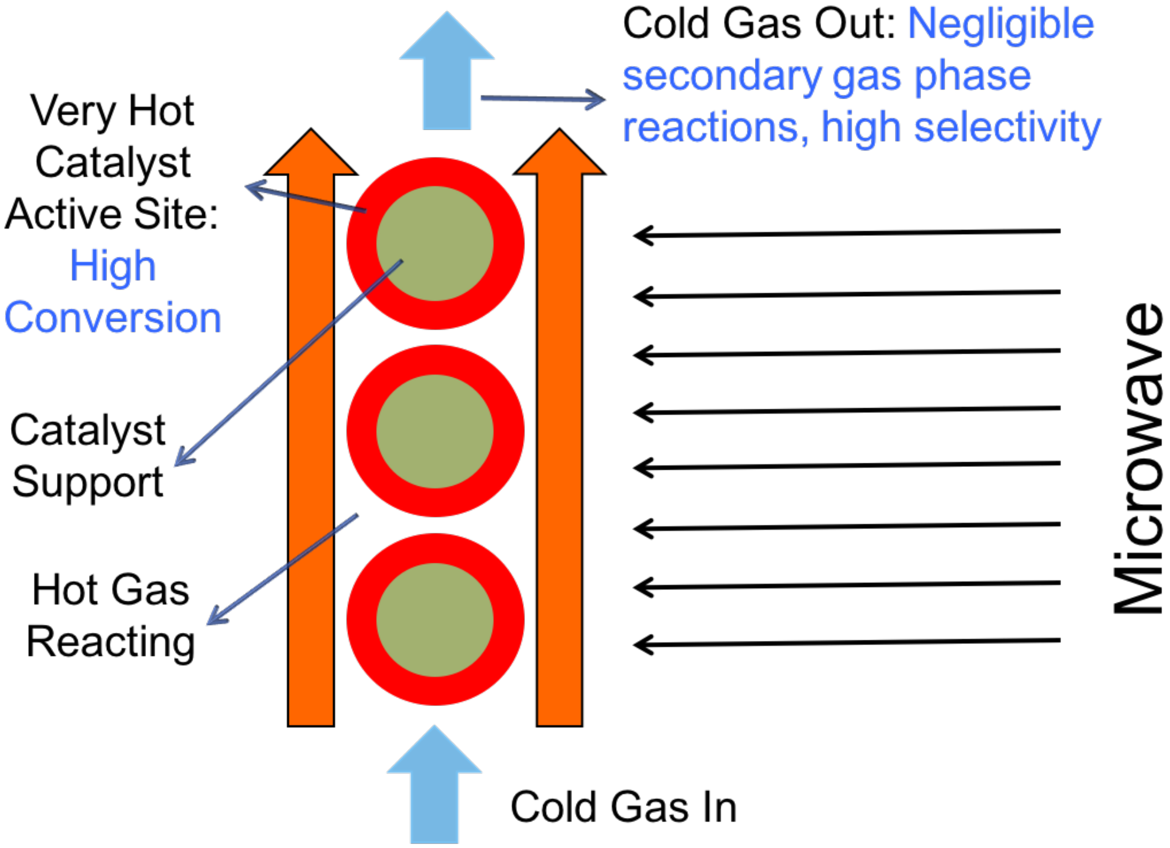

Extended Data Figure 7 – Effect of the Microwave Selective Heating Mechanism on the Gas-Solid Catalytic Reactions. While the gas enters the reactor at ambient temperature, due to insignificant dielectric properties it would not project microwave interaction. Concurrently, the catalyst active sites retain extremely high temperature due to the exceptional dielectric properties of the C-SiO_2 receptors. While the reaction proceeds on the active site, the temperature of the products and the reactants drastically drops while leaving the catalyst surface. Consequently, the gas does not acquire sufficient energy to evolve secondary gas-phase reactions. Consequently, microwave selective heating mechanism promotes catalytic reactions while restricting the production of secondary undesired by-products, simultaneously.

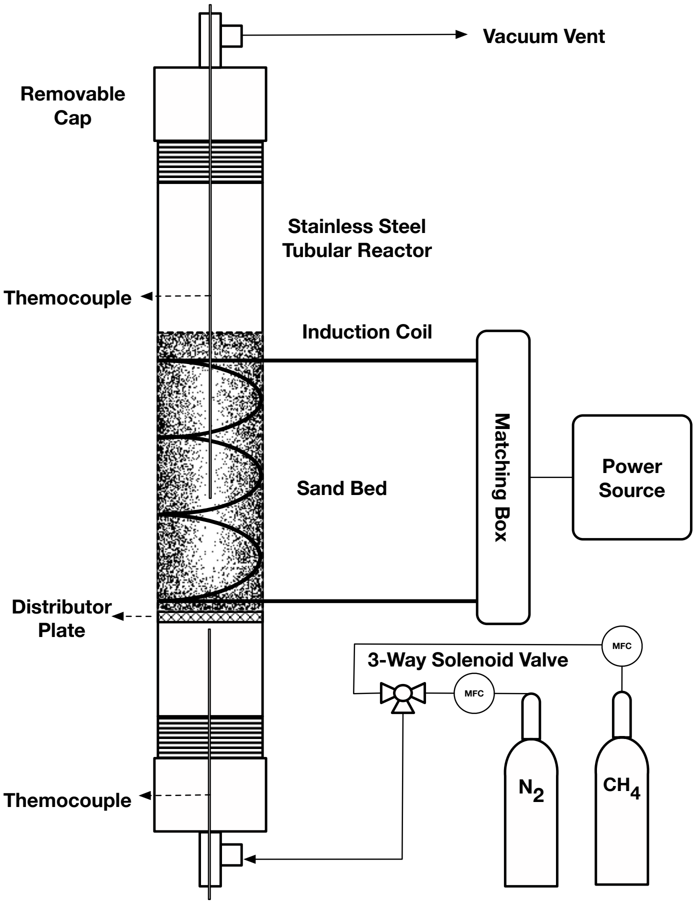

Extended Data Figure 8 - Induction Heating-Assisted Fluidized Bed CVD Experimental Setup. The induction heating mechanism, while generated by renewable electricity resources, provides an esteemed opportunity to develop environmental friendly and economically feasible chemical reactions. The CVD reactions were successfully performed in the present induction heating-assisted fluidized bed reactor or develop multiple grades of C-SiO_2 microwave receptors. The induction heating setup facilitated a comprehensive evaluation of the effect of the CVD operating conditions on the quality and performance of the developed particles.

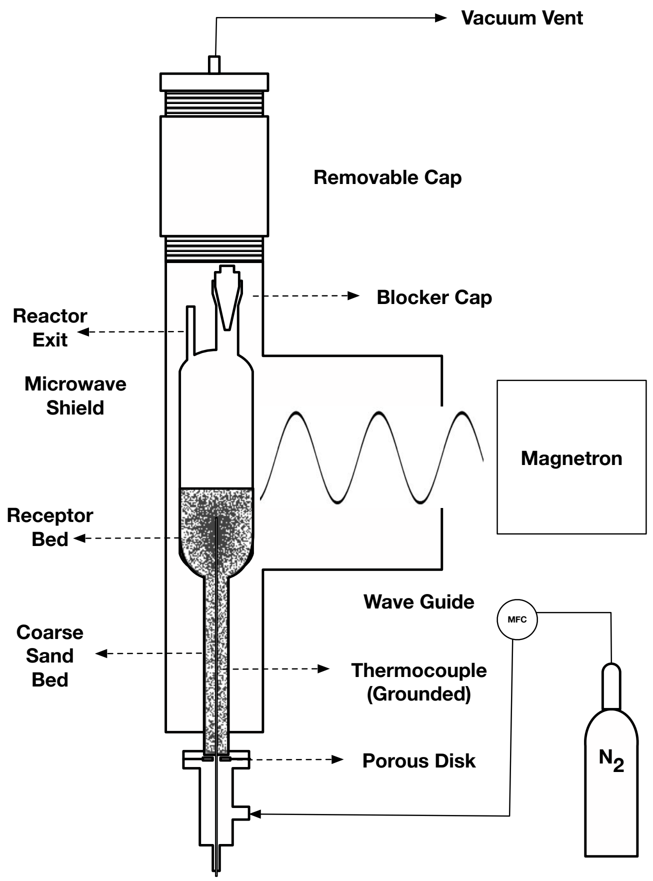

Extended Data Figure 9 – Microwave Heating Fluidized Bed Setup Diagram. Microwave heating mechanism coupled with the renewable electricity development is beneficial to optimize the productivity of the catalytic chemical reactions. The developed single-mode microwave-heated fluidized bed setup enabled us to study the effect of the CVD operating conditions and microwave power in the performance of the developed receptors, accordingly.

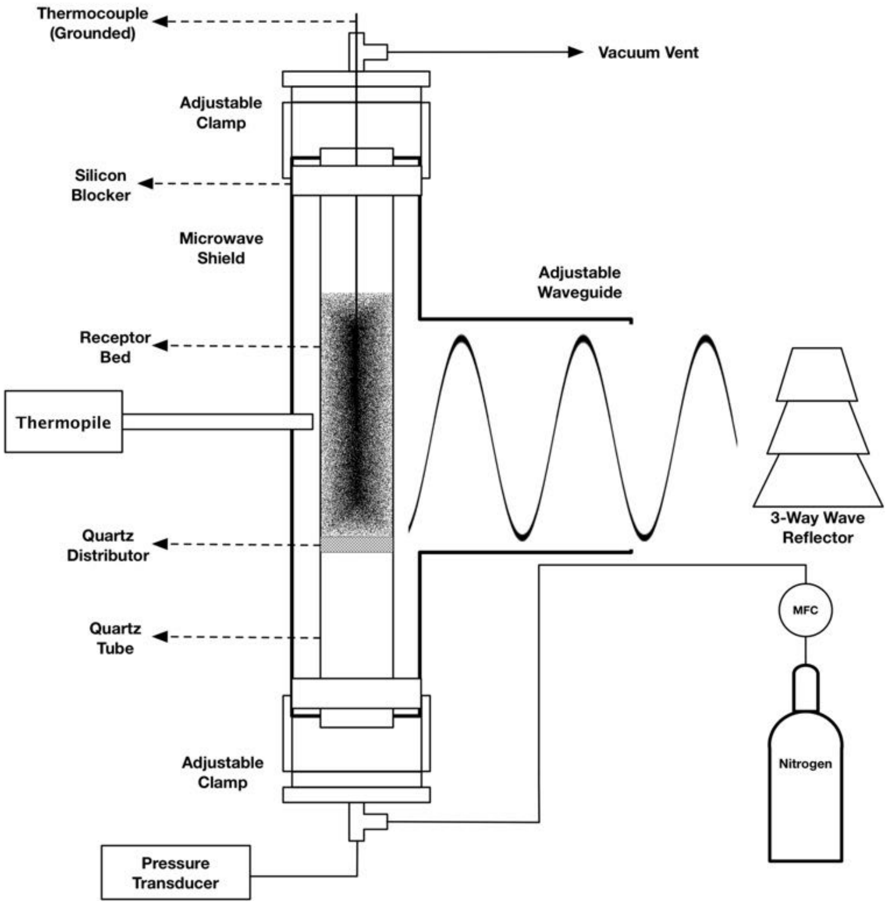

Extended Data Figure 10 – Schematic Diagram of the Microwave Heating-Assisted Fluidized Bed Apparatus. The measurement of the temperature in a microwave-heated setup assisting thermometric methods is complex due to the interaction of the metals with the electromagnetic field[56]. Consequently, thermopile, a radiometry temperature measurement method, was developed to monitor the temperature of the receptors while exposed to microwave irradiation. The voltage generated by the light capturing of the C-SiO_2 particles radiated while exposed to microwave was converted to temperature, correspondingly. The setup assisted us to study the effect of the operating conditions on the temperature gradient between the solids surface and the bed bulk and develop correlations to estimate the gas temperature, accordingly (see citation 25).

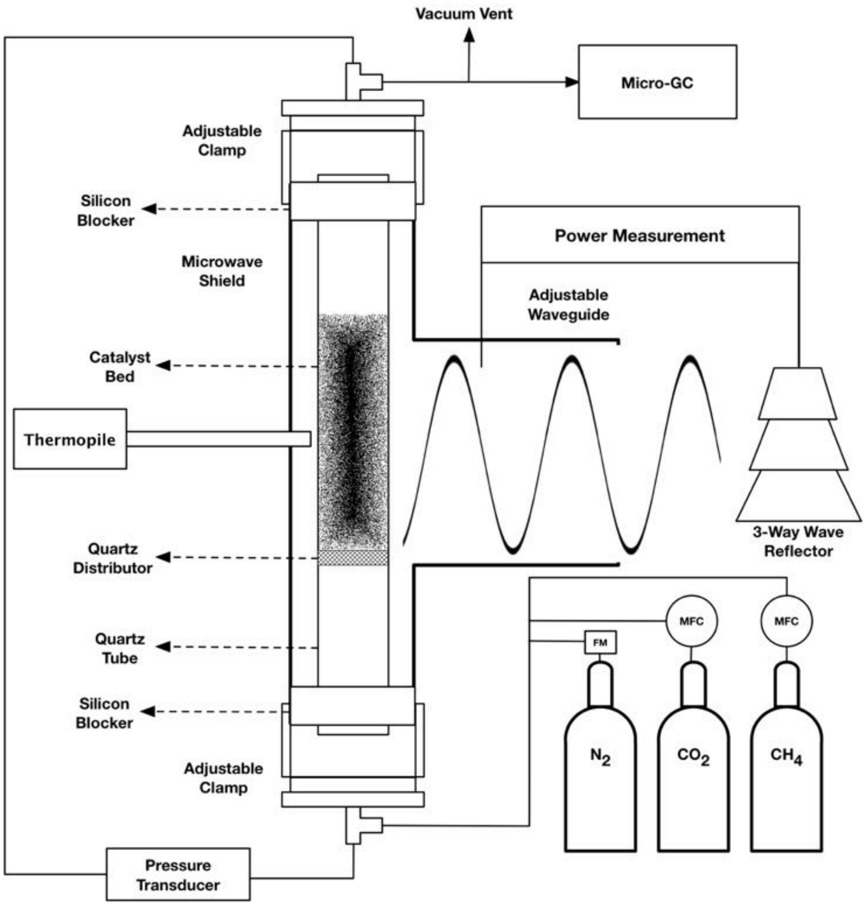

Extended Data Figure 11 – Schematic Demonstration of the DRM Microwave Heating Apparatus. The development of renewable electricity resources, namely, wind and solar power, has established a prominent opportunity for microwave heating reactions. Moreover, the selective microwave heating mechanism provides and exclusive prospect to address the catalytic reaction deficiencies. The temperature measurement of the solid bed particles was monitored by a thermopile, while the incident and reflected power was constantly regulated to enhance the performance of the microwave heating reaction. The evolved components were further analyzed by a micro-GC to determine the composition of the output and productivity of the reaction, correspondingly. The setup facilitated to investigate the effect of the microwave selective heating mechanism on the conversion of the reactant and selectivity of the product in the DRM process.

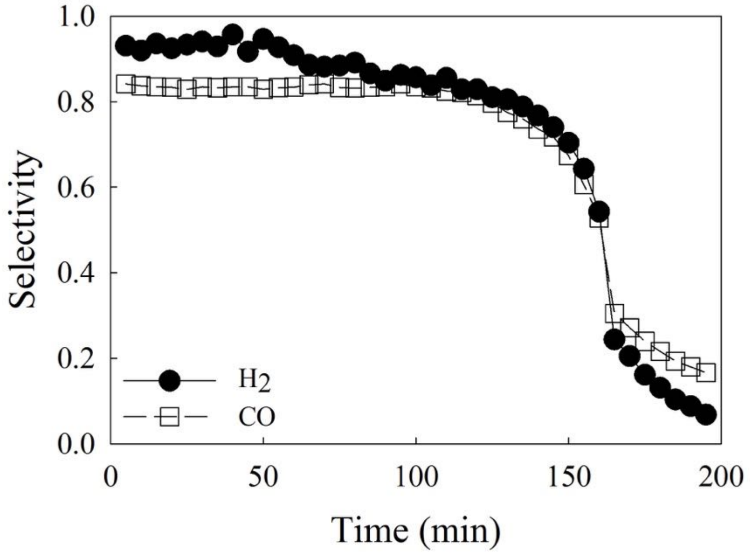

Extended Data Figure 12 – HiFUEL R110 Catalyst Deactivation as a Function of Time at 800°C to 900°C Operating Temperature Range and CO_2/CH_4=1:1. The catalyst deactivation occurs due to the deposition of the carbon species on the active sites. The carbon deposition is regarded as one of the major deficiencies of the methane conversion processes. While the catalyst performance was maintained stationary in the initial 150 minutes, impulsive deactivation occurred due to the uncontested methane decomposition process thereafter. In general, nickel base catalyst are prone to deactivation in association with the coking reactions while performing DRM reactions.

51 Latifi, M. & Chaouki, J. A novel induction heating fluidized bed reactor: Its design and applications in high temperature screening tests with solid feedstocks and prediction of defluidization state. *AIChE Journal* **61**, 1507-1523, doi:10.1002/aic.14749 (2015).
52 Ma, J. *et al.* Microwave-assisted catalytic combustion of diesel soot. *Applied Catalysis A: General* **159**, 211-228, doi:http://dx.doi.org/10.1016/S0926-860X(97)00043-4 (1997).
53 Vos, B., Mosman, J., Zhang, Y., Poels, E. & Bliek, A. Impregnated carbon as a susceptor material for low loss oxides in dielectric heating. *J Mater Sci* **38**, 173-182, doi:10.1023/a:1021138505264 (2003).
54 Uhlig, H. H. & Keyes, F. G. The Dependence of the Dielectric Constants of Gases on Temperature and Density. *The Journal of Chemical Physics* **1**, 155-159 (1933).
55 Gupta, M. & Wong, W. L. *Microwaves and metals*. (John Wiley & Sons, 2007).
56 Pert, E. *et al.* Temperature measurements during microwave processing: The significance of thermocouple effects. *Journal of the American Ceramic Society* **84**, 1981-1986, doi:10.1111/j.1151-2916.2001.tb00946.x (2001).
